# Analysis of hyaluronic acid concentration in rat vocal folds during estral and gravidic puerperal cycles

**DOI:** 10.1016/S1808-8694(15)30513-9

**Published:** 2015-10-18

**Authors:** José Eduardo de Sá Pedroso, Osíris Camponês do Brasil, João Roberto Maciel Martins, Helena Bociane Nader, Manuel de Jesus Simões

**Affiliations:** 1 Master and Doctor, Coordinator of the Outpatient Clinic of Larynx and Voice at UNIFESP-EPM; 2 Master and Doctor, Post-Graduation Instructor Professor at the Department of Otorhinolaryngology and Head and Neck Surgery at UNIFESP/EPM; 3 Master and Doctor, affiliated Professor at the Discipline of Molecular Biology at the Department of Biochemistry at UNIFESP-EPM; 4 Master, Doctor and Faculty Member, Full Professor at the Discipline of Molecular Biology at the Department of Biochemistry at UNIFESP/EPM; 5 Faculty Member, Associate Professor at the Discipline of Histology of the Departament of Morfology at UNIFESP/EPM

**Keywords:** vocal cords, hormones, larynx, hyaluronic acid

## Abstract

Hormone plays an important role in the larynx. Among other substances, vocal folds contain hyaluronic acid, which tissue concentration may vary according to hormone action.

**Aim:**

the objective of this study is to analyze hyaluronic acid concentration in the vocal folds during estral and gravidic-puerperal cycles.

**Study design:**

Experimental study.

**Materials and Methods:**

40 adult rats were divided into two groups. In the first group we used 20 rats to establish the concentration of hyaluronic acid during the estral cycle and in the second group, 20 animals were submitted to the same procedure but during the gravidic-puerperal cycle.

**Results:**

Variations in hyaluronic acid concentration was not observed during the estral cycle. In the gravidic puerperal cycle group, an increase in hyaluronic acid concentration was observed in the puerperal subgroup. Comparing the two groups of estral and gravidic-puerperal cycles, no difference was observed.

**Conclusions:**

In comparing all subgroups of estral and gravidic-puerperal cycles, an increase in hyaluronic acid concentration was noticed only in the puerperal phase.

## INTRODUCTION

Hormones have an important influence in the larynx. Laryngeal changes are not limited to the pubertal period, andropause and menopause; they occur throughout the individual's lifetime. Vocal changes in women, associated with sexual hormones, are very common in the clinical practice and have been investigated in some periods, such as: premenstrual, pregnancy, and menopause. According to Greene & Dalton^1^, during the premenstrual period, when the levels of estrogen and progesterone are lower, a mild thickening of vocal folds occurs. The hormone impact occurs not only on the genital tract, but also on the mucosae, muscles, bone tissues, larynx and brain cortex (Abitbol et al., 1999)^2^. The activity of progesterone and estrogen in the premenstrual period causes vasodilation with consequent increase in blood volume, resulting in vocal fold (VF) edema ^3^. The macroscopic changes that we observe in vocal folds due to these hormones reflect the alterations occurring at microscopic and ultrastructural levels. With the development of more sensitive methods, the substances participating in this phenomenon can be detected and analyzed.

The vibration of vocal folds depends on size and constituents of the lamina propria (LP), among other factors. The substances that comprise the extracellular matrix (ECM) in this region include glycosaminoglycans (GAGs) and fibrous proteins (Hirano, 1981)^4^. Small quantitative changes in the macromolecules forming the ECM are able to cause negative impacts in the biomechanical properties of the VFs. Hyaluronic acid (HA) is a GAG and one of the main components of the LP. Its influence in the biomechanical properties of VFs was demonstrated by Gray et al. (1999)^5^ and Chan et al. (2001)^6^. HA was first cited in the medical literature by Meyer & Palmer^7^, in 1934. They isolated a high molecular weight acid polysaccharide from bovine vitreous humor which was not sulfated. They proposed to name it hyaluronic acid, from “hyaloid” (vitreous), in addition to uronic acid.

Tateya et al. (2005)^8^ stated in an experiment using rats as animal models that their LP is more similar to that of humans, and this animal is more appropriate to be used in VF studies.

According to Santos & Ferrazoli^9^ (2005), the reproductive periods can be classified as continuous or seasonal estral cycles. The estral cycle is the period between the successive phases of sexual receptivity, usually called heat or estrus. The cycle can be monoestral (once a year) or polyestral (twice a year or more). Duration of the continuous estral cycles is species-specific: in rats, for example, it lasts four to five days; in guinea pigs and owes it lasts up to 16 days, and in cows and female pigs it lasts up to 21 days. The ovarian function phases were divided into stages which correlated with vaginal cytology. In female rats these phases are: proestrus, estrus, metaestrus and diestrus. Knowledge about the relation between vaginal cytology and ovarian function provided physiologists and biochemists with a powerful experimental instrument.

The objective of this study is to comparatively analyze the concentration of hyaluronic acid in vocal folds of female rats during the estral cycle and the pregnancy-puerperal cycle.

## MATERIALS AND METHODS

The study project was submitted to the Ethics Committee and it was approved under number 1234/06.

A total of 32 adult female rats (sp-wistar) weighing approximately 250 grams were used. The animals were confined in plastic cages with metal bars and maintained with food and water ad libitum with controlled temperature and luminosity.

In the first group, we used 16 female rats to determine the HA concentration in the estral cycle. These rats were selected from a total of roughly 60 female rats that underwent a colpocytological examination to determine the phase of the cycle they were.

The animal was held by a laboratory technician; a cotton swab impregnated with 0.9% saline solution was inserted in the vagina. After that, a slide smear was carried out, which was immediately analyzed using a light microscope with 40X magnification. The estral cycle phases were determined according to cell quantification: epithelial, cornified and leukocytes in the colpocytological examination. In cases of doubt about the estral cycle classification, the animal was not included in the subgroup.

A subgroup was considered complete when a total of four animals was reached (proestrus, metaestrus, estrus and diestrus), with a total of 16 animals in the group (P-proestrus, M-Metaestrus, E-Estrus and D-diestrus). The animals were immediately killed.

In the second group (also with 16 animals) the same experiment was carried out in the pregnancy-puerperal cycle. The animals were divided into subgroups of four animals and were killed on the 7th, 14th and 21st day of pregnancy and on the 14th day of puerperium. The animals were mated at night and, on the next morning the pregnancy diagnosis was confirmed with the finding of sperm in the vagina of female rats (Hamilton & Wolfe^10^, 1938); after being killed, the animals underwent laparotomy to check the presence of fetuses in the uterus.

The animals were assigned letters followed by Roman numbers according to their subgroups.

In all subgroups the animals were used for the study of HA concentration. Since the VFs were small, both folds had to be used for the biochemical procedures in the subgroups.

The animals were sacrificed with thiazine hydrochloride (Rompun®) and ketamine, with intraperitoneal injection of a lethal dose.

**Figure 1:**
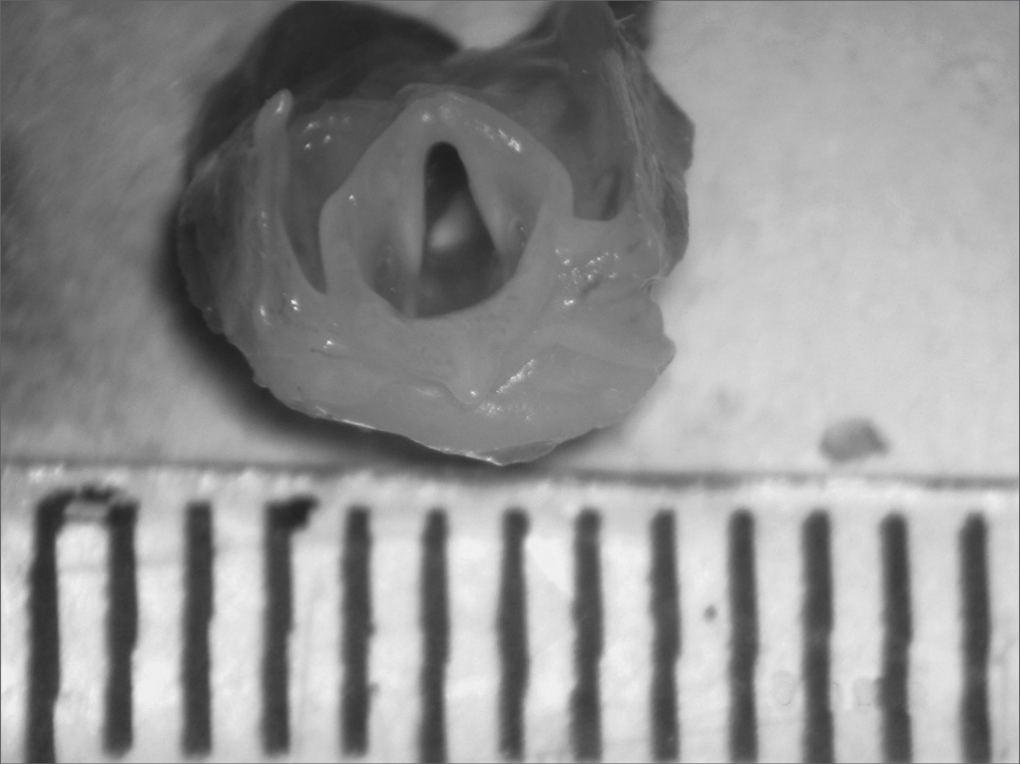
Detail of a female rat larynx.

After sacrifice, the vocal folds were removed with the use of a surgical microscope (DF Vasconcelos) in the dissection room. The animal was placed in supine position and hair shaving was not necessary. The incision was performed with a number 15 scapel in the midline sagittal plane at the level of the hyoid bone down to the beginning of abdominal region. Afterwards, the pre-thyroid muscles were dissected, the trachea was identified and separated from the esophagus, and subsequently we dissected the thyroid cartilage region. The specimen was removed with a lower limit at the level of second or third tracheal ring and upper limit at the level of the hyoid bone. Then, the specimen was “cleaned” so that only the larynx was kept (Figure 1), and placed in anatomical position and a posterior midline incision was performed in the entire surface. Two hemilarynges were available. The vocal folds were identified and a careful upper and lower incision in the VF was performed with a delicate scalpel to remove the vocal folds, avoiding the inclusion of arytenoid cartilages in the specimen.

**Figure 2:**
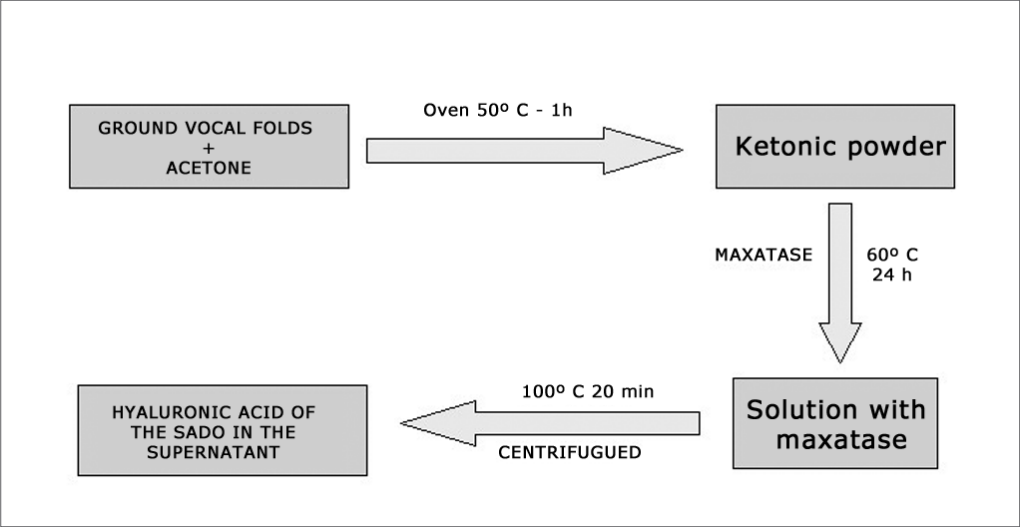
Diagram showing the sample preparation for a biochemical study.

The vocal folds of each animal were crushed and placed in ketone to remove lipid residues. Ketone was evaporated after a one-hour period in an oven at 50°C and the “ketonic powder” was obtained and weighed. The ketonic powder was added to maxatase (alkaline protease; Biocon do Brasil Industrial Ltda-RJ, Brazil) at the dose of 4 mg/mL in a Tris-HCL 0.05M buffer at pH of 8.0, added by NaCl 1 M (100 μl of enzyme for each 100 mg of dry powder). The mix was incubated at 60°C for 24 hours and, after this period, maxatase was inactivated by heating at 100°C for 20 minutes. After cooling, the samples were centrifuged and HA was measured in the supernatant (Figure 2).

A fluorimetric method was used to determine HA in biological fluids, by HA-binding “probes”. The probe is isolated from bovine nasal cartilage and is composed of the globular region of agregam (a proteoglycan formed by a protein skeleton to which the keratan sulfate and chondroitin sulfate chains bind) and the HA binding protein. The probe is used both immobilized in ELISA plates, similar to a capture antibody, and as a biotinylated probe, in the latter acting as a secondary labeled antibody.

To the ELISA plate with adsorbed probe,100 μl/well of standard HA solutions were added in several concentrations (0 to 500 μg/l), diluted in the assay buffer Tris-HCL 0,05 M, pH 7.75 and BSA 1% (bovine serum albumin), in addition to the samples obtained from tissues diluted in the same assay buffer (1:100) as triple copies. Incubation at 4°C was carried out for 12 hours and then the plate was rinsed with a rinsing buffer (Tris-HCL 0.05 M, pH 7.75) three times. After that, 100 μl from the probe (1mg/mL) 1:10000 were added in assay buffer. The plate was stirred for 2 hours and then washed nine times with a rinsing buffer. After this operation, 100 μl/well streptavidin labeled with europium diluted at 1:10,000 in an assay buffer were added to the plate. Streptavidin has affinity to the biotin conjugated to the probe. The plate was stirred for half an hour and then it was rinsed nine times with a rinsing buffer. Lastly, with the purpose of releasing the europium bound to streptavidin, an enhancement solution was added, 280μl/well; it was agitated for five minutes and the free europium in the plate was read in a fluorimeter. The result was obtained in ng/ml.

The subgroup results were submitted to analysis of variance (ANOVA) test. The ANOVA test is indicated to compare three or more groups of data with numerical measurement. The samples are independent and/or paired and they shows if the groups present different means. It is possible to test more than one effect with a single model.

## RESULTS

Table 1 shows the comparison of results between subgroups in the estral cycle group; whereas Table 2 displays the comparison between subgroups in the pregnancy-puerperal cycle group, and Table 3 the total comparison between subgroups in the estral cycle group and between subgroups in the pregnancy-puerperal cycle group.

**Table 1:** Comparison between subgroups in the estral group per measurement of μ HA / g dry tissue.

	Estrus	Diestrus	Metaestrus	Proestrus
Mean	95,68	100,40	85,38	110,30
Standard deviation	26,88	12,97	17,52	17,49
N	4	4	4	4

ANOVA (p) equals; 0.372 Non-significant. There was no difference between the subgroups regarding the measurement evaluated.

**Table 2:** Comparison between subgroups in the group pregnancy-puerperal on the 7th, 14th, 21st and 14th day of puerperium (P) according to the measurement of μ HA / g dry tissue.

	7TH DAY	14TH DAY	21ST DAY	P
Mean	84,98	84,45	76,74	250,15*
Standard deviation	16,12	36,35	9,07	71,80
N	4	4	4	4

* Significant. For multiple comparisons, group P had higher responses than other groups

**Table 3:** Total comparison between subgroups in group Estral (E, D, M and P) and subgroups in group Pregnancy (7, 14, 21 and P) per measurement of μ HA /g dry tissue.

		ESTRAL				PREGNANCY		
	E	D	M	P	7	14	21	P
Mean	95,68	100,40	85,38	110,30	84,98	84,45	76,74	250,1*
Standard deviation	26,88	12,97	17,52	17,49	16,12	36,35	9,07	71,80
N	4	4	4	4	4	4	4	4

## DISCUSSION

There is great difficulty to study - from the biochemical point of view- the VF of female corpses as to the hormonal phase, since the medical charts usually do not contain data about the menstrual cycle and medications used.

Rabbits and dogs were used in experiments with VFs (Rousseau^11^ et al., 2003; Rousseau^12^ et al., 2004), but, according to Tateya et al. (2005)^8^, the rat is a better model since the LP of its VFs has features similar to humans; it can be divided into

layers, with the deep layer containing more collagen fibers than the superficial layer. Additionally, in rats, there is extensive genetic information and, since the animal has a short lifecycle, it is possible to investigate several cycles. Due to its size and fertility, it is a more economically viable model. The cartilage portion of the larynx is about fivefold larger in rats than in humans, which can be pointed as a disadvantage in this model.

When establishing the time interval for animal sacrifice in the pregnancy-puerperal cycle, we chose performing it every seven days because, since the pregnancy in rats lasts about 21 days, we are dividing this period into three equal phases. We choose the 14th day of puerperium for sacrifice since weaning starts on this day and the female rat would have the same stimuli as in the estral cycle again.

The methods used to assess HA in other studie are indirect, as shown by Pontes^13^ et al. (1989), Hammond^14^ et al. (1997) and Butler^15^ et al. (2003), in which reagents for GAGs and proteins are used, and then testicular hyaluronidase is utilized to differentiate where HA was present by comparison or subtraction. However, this hyaluronidase is not specific for HA, and it can also break chondroitin sulfate. Therefore, in these studies, other substances may be included besides HA.

The method used to detect hyaluronic acid concentration is one of the most sensitive described in the literature. A specific hyaluronidase for HA is used and, in the fluorimetric method, a HA-binding protein is applied, isolated from the bovine cartilage that specifically recognizes HA.

Likar & Likar^16^ (1964), in their study of bovine breast cells and uterine wall cells, observed increased levels of mucopolysaccharide and, together with it, the hyaluronate fraction, according to the phase of the estral cycle. During the follicular phase (diestrus, proestrus and estrus), there was an increase in estrogen and a gradual rise in hyaluronate, which after ovulation (luteal phase) decreases in size again. Pontes^13^ et al. (1989) and Cubas^17^ (2001) conducted research in which HA was quantified in the nasal mucosa and uterine cervix of female rats, respectively, and both observed that there was no variation in the quantity of HA in these tissues during the estral cycle. In the investigation carried out by Likar & Likar^16^ (1964), the animal model was different and the technique for HA quantification was also diverse. These factors probably culminated with diverging results compared to ours.

In our investigation no significant variation in HA concentration in vocal folds of female rats during the estral cycle was observed. This result confirms the findings published by Pontes^13^ et al. (1989) and Cubas^17^ (2001) who, in their investigations, used the same animal model, but in different tissues; the technique that we used to measure HA was the same as that used by the second author.

Kofoed^18^ et al. (1972) showed that variation in HA content in the uterus of female rats is influenced by hormones. Estrogen would cause a decrease in HA concentration; therefore, one would expect that its concentration would vary during the estral cycle, since estrogen has its peak during the follicular phase and decreases again right after ovulation. However, the author did not perform his investigation with the physiological variation of hormones, having castrated and administered different doses of estradiol benzoate and, maybe for this reason, may have encountered a more evident variation in his results.

Pontes^13^ et al. (1989) observed in their sample the existence of a non-sulfated acid mucopolysaccharide, possibly HA, which decreased with pregnancy progression. In the invetigation by Cabrol^19^ et al. (1985), the tissues studied from urogenital organs of animals in the gestational period, are more sensitive to hormone action, since with pregnancy progression there is a decrease in estrogen and, as shown by Kofoed^18^ et al. (1972), this would lead to increased amount of HA. Rajabi^20^ et al. (1992) and Kobayashi^21^ et al. (1999) studied HA in the serum of guinea pigs and in women, respectively, and revealed an increase of HA as pregnancy progresses. This is likely to occur due to increased mobilization of HA in the tissues.

In our sample no variation in HA concentration was observed during pregnancy. It was expected to be found, since Newman^22^ et al. (2000) showed that VF has receptors for gonadotrophic hormones. To explain this data, we can suppose that the hormones would have a different action in the urogenital organ tissues, which participate more actively in the gestational process increasing the HA concentration and mobilizing the HA through the blood, while in other tissues its action would be less evident.

Pontes^13^ et al. (1989) observed that in the puerperium the non-sulfated acid mucopolysaccharide reappeared. In our investigation, a significant increase in HA concentration was found in this period. It was expected that, after delivery, with the hormones returning to normal levels, HA would also present the previously observed levels. According to Rajabi^20^ (1992), the HA levels in the serum of female guinea pigs returned to normal two days after delivery. We observed that, even comparing with the same period of the estral cycle, i.e., not during pregnancy, HA concentration was significantly higher in the puerperium, which makes us suppose that, with breastfeeding, other substances may act and cause changes in the HA dynamics.

Maybe our sample was small and to detect more substantial changes a larger sample would be necessary. On the other hand, in our experiment, the method performed is one of the most sensitive to analyze HA concentration.

Our study showed that the HA concentration may vary in the vocal folds and that this phenomenon is probably influenced by several factors. The importance of this substance in the phonatory function inspires us to perform deeper investigations in this field towards a possible clinical correlation in the future.

## CONCLUSIONS

From the analysis of hyaluronic acid concentration on the vocal folds of female rats during the estral cycle and pregnancy-puerperium cycle we can conclude that:

1. There is no variation in hyaluronic acid concentration during the estral cycle
2. In puerperium there is an increase in hyaluronic acid concentration
3. Comparing all subgroups of the estral cycle and the pregnancy-puerperium cycle, only the puerperium presents increased hyaluronic acid concentrations.

## References

[1] Grene RJ, Dalton K (1953). The Premenstrual Syndrome. Brit Med J..

[2] Abitbol J, Abitbol P, Abitbol B (1999). Sex hormones and Female voice. J Voice..

[3] Satalof RT, Emerich KA, Hoover CA, Satalof RT (1997). Professional voice: the science and art of clinical care.

[4] Hirano M. Structure of the vocal fold in normal and disease states-anatomical and physical studies. In: Ludlow CL, Hard MO, editors. American Speech Language-Hearing Association. Proceedings of the conference on the assessment of vocal pathology; 1981; Rockville, Maryland 1981. p. 11–30.

[5] Gray SD, Titze IR, Cha R, Hammond TH (1999). Vocal Fold Proteoglycans And Their Influence on Biomechanics. Laryngoscope..

[6] Chan RW, Gray SD, Titze IR (2001). The Importance of hyaluronic acid in vocal fold biomechanics. Otolaryngol Head Neck Surg..

[7] Meyer K, Palmer JW (1934). The Polysaccharide of Vitreous Humor. J Biol Chem..

[8] Tateya T, Sohn JH, Tateya I, Bless DM (2005). Histologic Characterization of Rat Fold Scarring. Ann Otol Rhinol Laryngol..

[9] Santos AV, Ferrazzoli MO. Ciclo estral nos animais. [Site na internet]: Artigos científicos. Disponível em: http//www.redevet.com.br/artigos/estral1.htm. Acessado em abril de 2005

[10] Hamilton JB, Wolf JM (1938). The effect of male hormone substances upon birth and prenatal development in the rat. Anat Rec..

[11] Rousseau B, Hirano S, Scheidt TD, Welham NV, Thibeault S, Chan RW, Bless DM (2003). Characterization of Vocal Fold Scarring in a Canine Model. Laryngoscope..

[12] Rousseau B, Hirano S, Chan RW, Welham NV, Thibeault S, Ford CN, Bless DM (2004). Characterization of Chronic Vocal Fold Scarring in a Rabbit Model. J Voice..

[13] Pontes PAL, Simões MJ, Merzel J (1989). Histoquimic detection of glicoproteins and glycosaminoglycans on respiratory mucose of albine mouses during estral cycle and pregnant. Rev Bras Biol..

[14] Hammond TH, Zhou R, Hammond EH, Pawlak A, Gray SD (1997). The intermediate layer: a morphologic Study of the elastin and hyaluronic acid constituents of normal human vocal fold. J Voice..

[15] Butler JE, Hammond TH, Gray SD (2001). Gender-related differences of hyaluronic acid distribution in the human vocal fold. Laryngoscope..

[16] Likar IN, Likar JL (1964). Acid mucopolyssacarides and mast cells in the bovine uterus at different stages of the sexual cycle. Acta Endocr..

[17] Cubas JMC. Análise histomorfológica e caracterização dos glicosaminoglicanos no colo uterino de ratas durante o ciclo estral. [Dissertação] São Paulo (SP): Universidade Federal de São Paulo; 2002.

[18] Kofoed AJ, Houssay AB, Tocci AA, Curbelo HM (1972). Efects of oestrogens upon glycosaminoglicans in the uterus of rats. Acta Endocr..

[19] Cabrol D, Dallot E, Cedard L, Sureau C (1985). Pregnancy-related changes in the distribution of glycosaminoglycans in the cervix and corpus of the human uterus. Eur J Obstet Gynecol Reprod Biol..

[20] Rajabi RM, Quillen EW, Nuwayhid BS, Brandt R, Poole AR (1992). Circulating hyaluronic acid in nonpregnant, pregnant, and postpartum guinea pigs: Elevated levels observed at parturition. Am J Obstet Gynecol..

[21] Kobayashi H, Sun GW, Tanaka Y, Kondo T, Terao T (1999). Serum hyaluronic acid levels during pregnancy and labor. Obstet Gynecol..

[22] Newman RS, Butler J, Hammond EH, Gray S (2000). Preliminary Report on Hormone Receptors in Human Vocal Fold. J Voice..

